# Supplementary material for: Polar localization of putative phospholipid transporters in Escherichia coli
Source: mBio. 2026 Feb 9;17(3):e03481-25. doi: 10.1128/mbio.03481-25 (PMC12977461; doi:10.1128/mbio.03481-25)
Supplement: Supplemental information — Figures S1-S9; Tables S1-S3; movie legends. [file mbio.03481-25-s0001.pdf]

Supplemental Material

Polar localization of putative phospholipid transporters in *Escherichia coli*

Wee Boon Tan[1,2,#], Zhi-Soon Chong[1,#], Jacob Wye Meng Cheong[1,#], Jiang Yeow[1], and Shu-Sin Chng[1,2,*]

Affiliations:

[1]Department of Chemistry, National University of Singapore, Singapore 117543.

[2]Singapore Center for Environmental Life Sciences Engineering, National University of Singapore (SCELSE-NUS), Singapore 117456.

[#] These authors contributed equally

* To whom correspondence should be addressed.

E-mail: chmchngs@nus.edu.sg

ORCID ID:

Wee Boon Tan: 0000-0002-5055-4517

Shu-Sin Chng: 0000-0001-5466-7183

Jiang Yeow: 0000-0001-5384-7211

This PDF file includes:

Figure S1 to S9
Table S1 to S3

As separate files:

Supplemental Movie 1 to 4

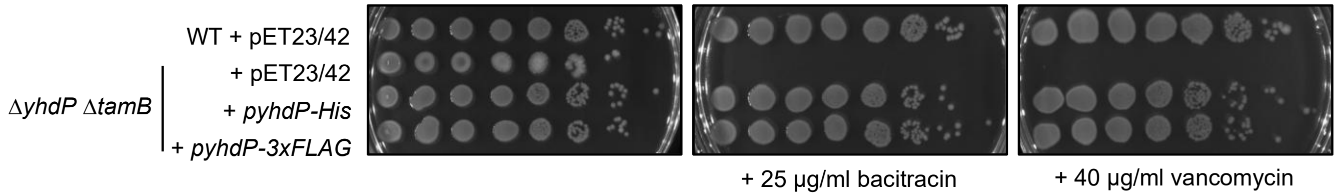

Figure S1. YhdP-His and YhdP-Flag are functional. Efficiency of plating (EOP) of MG1655 WT and Δ*yhdP* Δ*tamB* strains, expressing either YhdP-His or YhdP-FLAG on LB agar plates supplemented with bacitracin (25 µg/ml) or vancomycin (40 µg/ml) at 37°C. Cells with defective outer membrane (OM) barrier function become permeable to vancomycin and bacitracin, drastically sensitizing them towards these molecules. Δ*yhdP* Δ*tamB* double deletions result in OM barrier defects and sensitivity towards bacitracin and vancomycin (ref. 1); functional construct of YhdP restores the resistance.

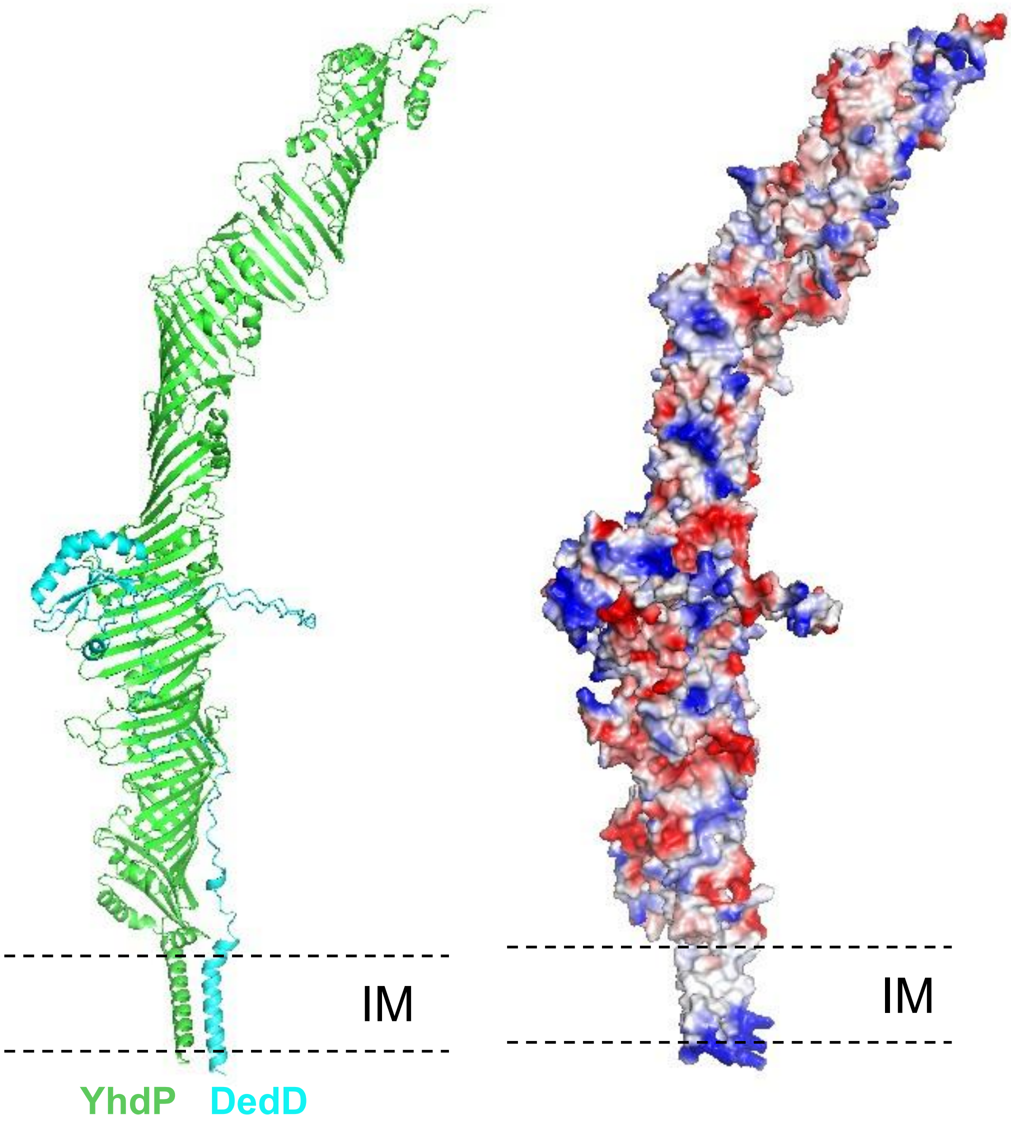

Figure S2. AlphaFold3 prediction of YhdP-DedD heterodimer. Possible structural model of a YhdP-DedD complex was predicted using the AlphaFold3 web server by inputting the YhdP and DedD protein sequences from MG1655. Resulting model of moderate confidence (ipTM = 0.6) suggests interaction interfaces at the transmembrane and periplasmic regions.

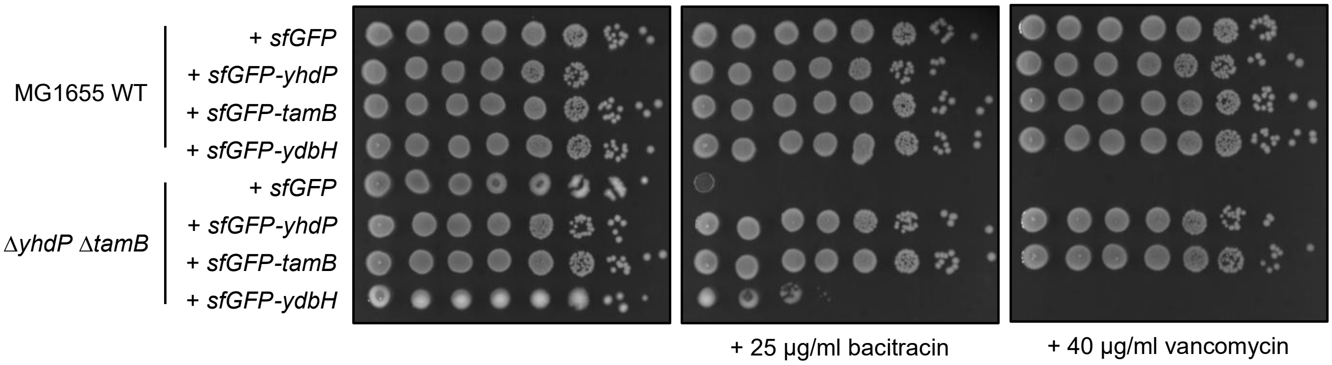

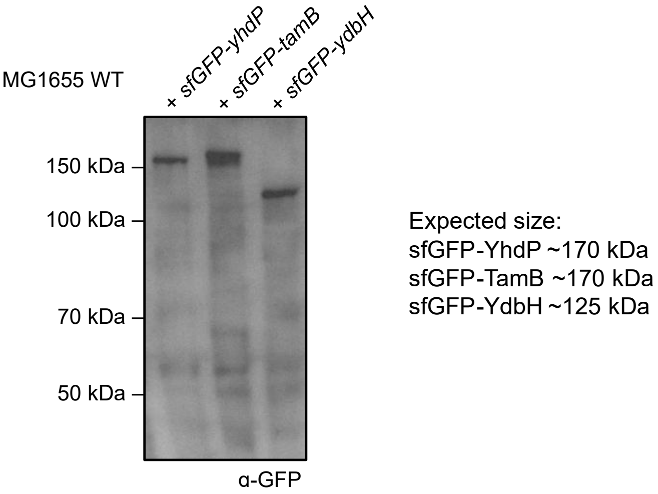

Figure S3. sfGFP fusions of YhdP, TamB, and YdbH are functional. (*Top*) EOP of MG1655 WT and Δ*yhdP* Δ*tamB* strains, expressing either sfGFP, sfGFP-YhdP, sfGFP-TamB, or sfGFP-YdbH, on LB agar plates supplemented with bacitracin (25 μg/ml) or vancomycin (40 μg/ml) at 37°C. Functional construct of YhdP or TamB restores resistance to bacitracin and vancomycin in the Δ*yhdP* Δ*tamB* double deletions strain at the concentration tested. Expression of functional YdbH is known to rescue outer membrane defects partially (ref. 3). (*Bottom)* Immunoblots of the whole cell lysate of the indicated strains using α-GFP antibody.

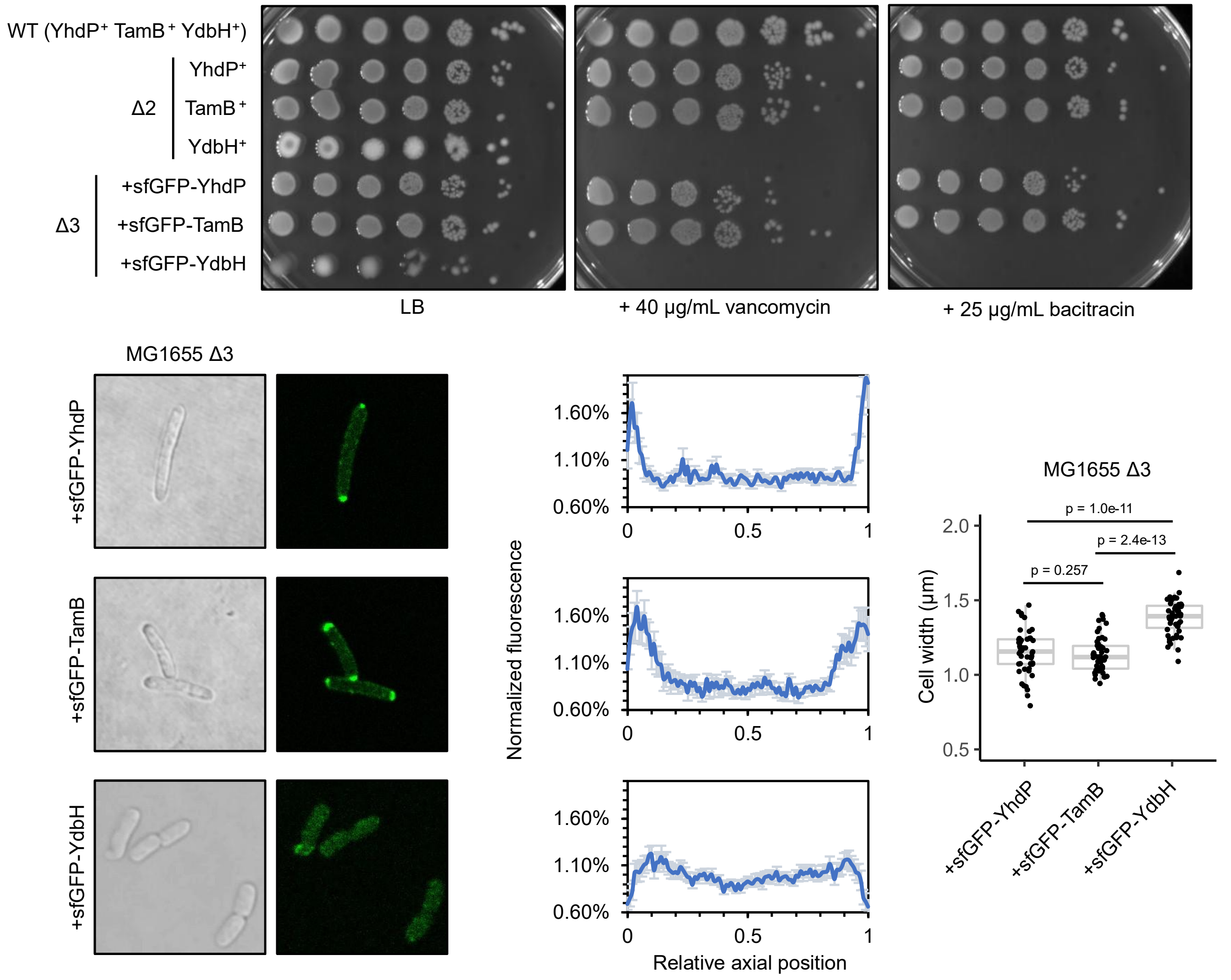

Figure S4 Either one of sfGFP-YhdP, -TamB, or -YdbH is sufficient as the sole transporter to support growth. (*Top*) EOP of MG1655 Δ3 (Δ*yhdP*::*frt* Δ*tamB*::*kan* Δ*ydbH*::*frt*) strains, expressing either sfGFP-YhdP, -TamB, or -YdbH from the respective pET23/42 plasmids, on LB agar plates supplemented with bacitracin (25 µg/ml) or vancomycin (40 µg/ml) at 37°C. The different plasmids were first transformed into an unmarked Δ*yhdP* Δ*ydbH* strain, where *tamB* was subsequently deleted using P1 transduction. Sensitivity to vancomycin and bacitracin of the Δ3 strains with either sfGFP fusion protein are identical to their corresponding Δ2 strain (with either native *yhdP*, *tamB*, or *ydbH* not deleted from the chromosome). (*Bottom*) Fluorescence microscopy images, distribution of the fluorescence along the long axis of cells (error bars represent 95% confidence intervals), and quantification of the cell width of the Δ3 strains (statistical significance tested with Wilcoxon-ranked-sum test).

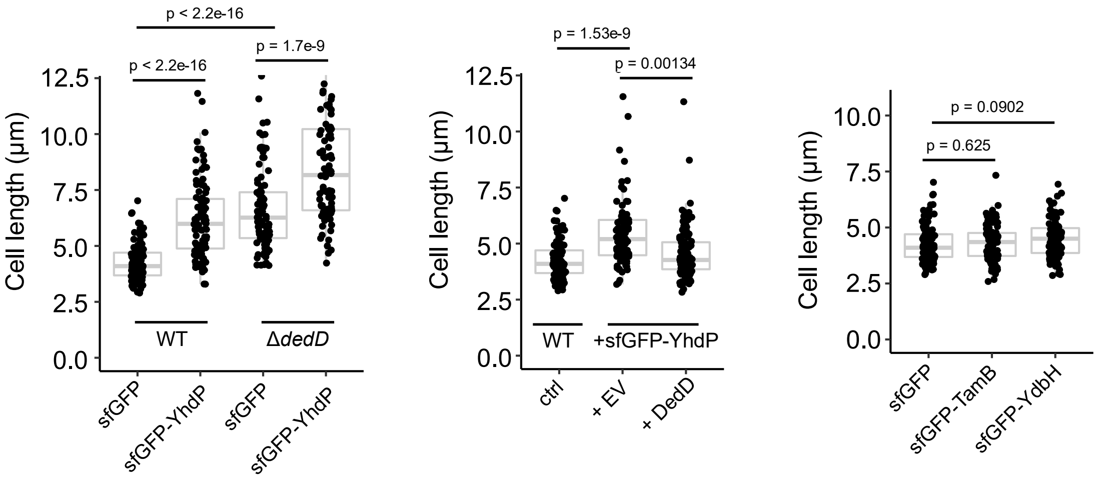

Figure S5. Expression of sfGFP-YhdP causes mild division defects. Cell length of the indicated strains was measured from the same samples used in Figure 2. Expression of sfGFP-YhdP (*left*), but not sfGFP-TamB or sfGFP-YdbH (*right*) resulted in longer cells, indicative of division defects. Expression of additional DedD reversed this morphological defect (*middle*). Statistical significance tested with Wilcoxon-ranked-sum test.

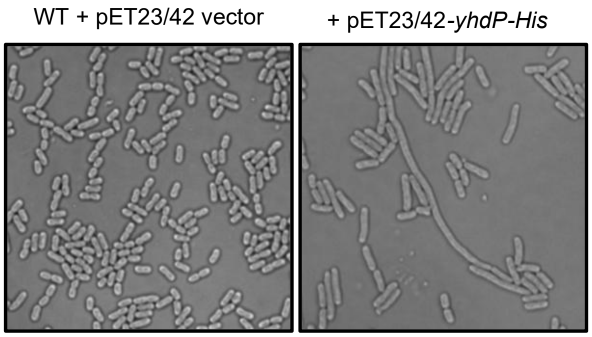

Figure S6. Expression of YhdP-His also causes cell division defects. Differential interference contrast microscopy images of WT strains containing either the pET23/42 empty vector or expressing YhdP-His from the same vector.

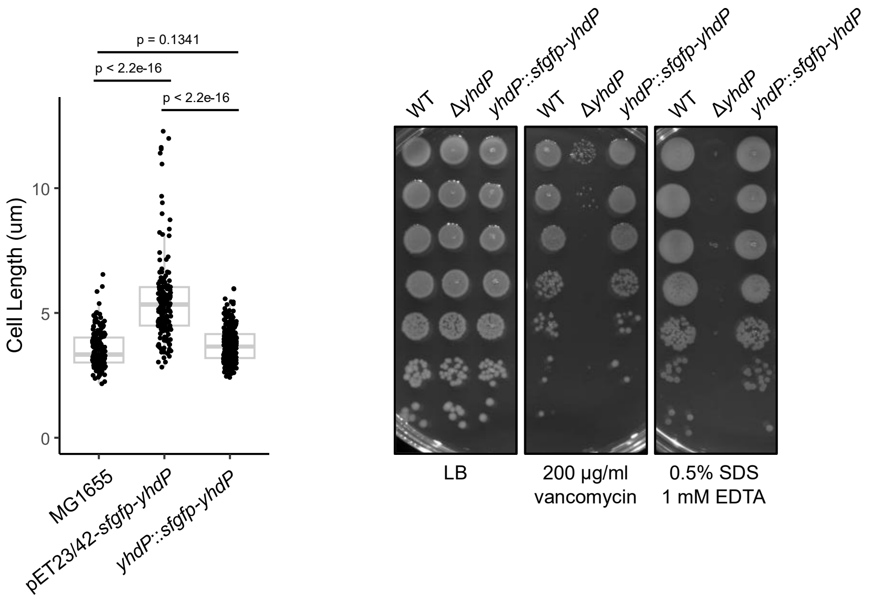

Figure S7. Chromosomally-encoded sfGFP-YhdP is functional. (*left*) Cell length quantification (statistical significance tested with Wilcoxon-ranked-sum test) of the chromosomally encoded *yhdP*::*sfgfp-yhdP* strain. (*right*) EOP of MG1655 *yhdP*::*sfgfp-yhdP* strain in comparison with WT and Δ*yhdP* on LB agar plates supplemented with vancomycin (200 µg/ml) or 0.5% SDS 1 mM EDTA at 37°C.

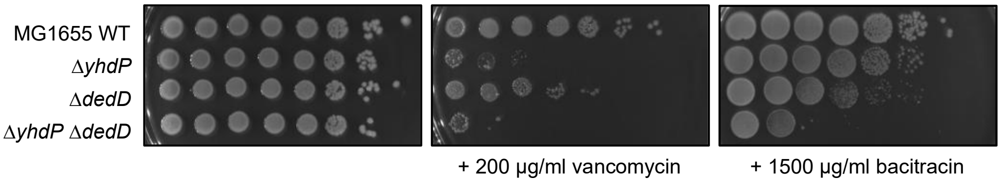

Figure S8. Deletion of *dedD* does not result in OM defects similar to when YhdP is removed. EOP of MG1655 WT, Δ*yhdP,* Δ*dedD,* and Δ*yhdP* Δ*dedD* strains on LB agar plates supplemented with bacitracin (1500 μg/ml) or vancomycin (200 μg/ml) at 37°C.

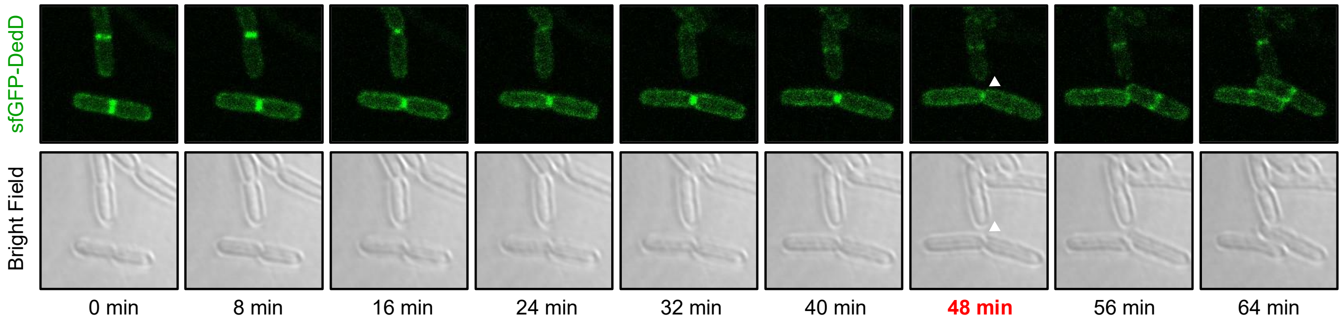

Figure S9. Fluorescence time-lapse microscopy tracking the fluorescence signal of sfGFP-DedD. Images were originally taken at 2-minute intervals (see Supplemental Movies 4) but presented here at 8-minute intervals between frames. White arrow(s) and red timepoint indicate completion of cell division and clear separation of daughter cells.

Table S1. Strain list

Strain	Reference
MG1655	WT
MG1655 Δ*dedD*::*kan*^{R}	This study
MG1655 Δ*yhdP*::*frt* Δ*tamB*::*kan*^{R}	This study
MG1655 Δ*yhdP*::*frt* Δ*ydbH*::*frt*	This study
MG1655 Δ*ydbH*::*frt* Δ*tamB*::*kan*^{R}	This study
MG1655 Δ3 [Δ*yhdP*::*frt* Δ*ydbH*::*frt* Δ*tamB*::*kan*^{R}] *	This study

***** MG1655 Δ3 genotype were constructed and maintained in the presence of pET23/42-*sfgfp-yhdP,* pET23/42-*sfgfp-tamB*, or pET23/42-*sfgfp-ydbH* plasmids.

Table S2. Plasmid list

Strain	Resistance	Remarks
pET23/42	Ampicillin	Empty vector (ref. 2)
pBAD33	Chloramphenicol	Empty vector (ref. 3)
pET23/42-*yhdP-His*	Ampicillin	Pull down
pET23/42-*yhdP-3xFLAG*	Ampicillin	Reciprocal pull down
pET23/42-*sfgfp*	Ampicillin	Localization experiment
pET23/42-*sfgfp-dedD*	Ampicillin	Localization experiment
pET23/42-*sfgfp-yhdP*	Ampicillin	Localization experiment
pET23/42-*sfgfp-tamB*	Ampicillin	Localization experiment
pET23/42-*sfgfp-ydbH*	Ampicillin	Localization experiment
pBAD33-*His-dedD*	Chloramphenicol	Reciprocal pull down
pBAD33-3x*FLAG-dedD*	Chloramphenicol	Rescue of cell division defects

Table S3. Primers list

Primer name	Nucleotide sequence	Remarks
sfgfp-yhdP5	gagctctacaaaGGATCCAGGCGATTGCCGGGGAT	Amplify *yhdP* from genomic DNA, with flanking homology to *sfgfp* (5') and pET23/42 backbone (3'), introduce two a.a. (glycine-serine between last a.a. of sfGFP and second a.a. of YhdP
sfgfp-yhdP3	GTGGTGGTGCTCGAGtcaTTGCGCTTTTTCTTTACG	
pET2342sfgfp-yhdP5	CGTAAAGAAAAAGCGCAAtgaCTCGAGCACCACCAC	Amplify pET23/42sfgfp backbone from pET23/42 (*sfgfp* inserted between NdeI and XhoI sites), with flanking homology to *yhdP*
pET2342yhdP3	ATCCCCGGCAATCGCCTGGATCCtttgtagagctc	
sfgfp-tamB5	gagctctacaaaGGATCCAGTTTATGGAAAAAAATC	Amplify *tamB* from genomic DNA, with flanking homology to *sfgfp* (5') and pET23/42 backbone (3'), introduce two a.a. (glycine-serine between last a.a. of sfGFP and second a.a. of TamB
sfgfp-tamB3	GTGGTGGTGGTGCTCGAGctaAAACTCGAACTGATAGAGC	
pET2342sfgfp-tamB5	GCTCTATCAGTTCGAGTTTtagCTCGAGCACCACCACCAC	Amplify pET23/42sfgfp backbone from pET23/42 (*sfgfp* inserted between NdeI and XhoI sites), with flanking homology to *tamB*
pET2342sfgfp-tamB3	GATTTTTTTCCATAAACTGGATCCtttgtagagctc	
sfgfp-ydbH5	gagctctacaaaGGATCCCTGGGTAAATATAAAGCCG	Amplify *ydbH* from genomic DNA, with flanking homology to *sfgfp* (5') and pET23/42 backbone (3'), introduce two a.a. (glycine-serine between last a.a. of sfGFP and second a.a. of YdbH
sfgfp-ydbH3	GTGGTGGTGGTGCTCGAGtcaTTGTTTTTCCTCACACTC	
pET2342sfgfp-ydbH5	GAGTGTGAGGAAAAACAAtgaCTCGAGCACCACCACCAC	Amplify pET23/42sfgfp backbone from pET23/42 (*sfgfp* inserted between NdeI and XhoI sites), with flanking homology to *ydbH*
pET2342sfgfp-ydbH3	CGGCTTTATATTTACCCAGGGATCCtttgtagagctc	
sfgfp-dedD5	gagctctacaaaGGATCCGCAAGTAAGTTTCAGAATCGG	Amplify *dedD* from genomic DNA, with flanking homology to sfgfp (5') and pET23/42 backbone (3'), introduce two a.a. (glycine-serine between last a.a. of sfGFP and second a.a. of DedD
sfgfp-dedD3	GCCTAGGTATTAATCAAttaATTCGGCGTATAGCCC	
pET2342sfgfp-dedD5	GGGCTATACGCCGAATtaaTTGATTAATACCTAGGC	Amplify pET23/42sfgfp backbone from pET23/42 (*sfgfp* inserted between NdeI and XhoI sites), with flanking homology to *dedD*
pET2342sfgfp-dedD3	CCGATTCTGAAACTTACTTGCGGATCCtttgtagagctc	
yhdP::cat-tse2-5	TTTTTGAGTCACATTTTTAGCAGACAAGGAGTGACGGgtgCTTCATTTAAATGGCGCGCC	Amplify *cat-tse2* (positive and negative selection marker) with flanking homology arms to yhdP locus, product transformed into MG1655 with lambda red helper plasmid for marking the *yhdP* locus
yhdP::cat-tse2-3	GCGCGGCTCCAGTAAGCAGTAAAATCCCCGGCAATCGCCTCATATGAATATCCTCCTTAG	
H1-sfgfp-yhdP5	GGGTTTTTGAGTCACATTTTTAGCAGACAAGGAGTGACGGatgtctaaaggtgaagaac	Amplify *sfgfp-yhdP* with homology arms to *yhdP* locus, product transformed into MG1655 with lambda red helper plasmid for marking the *yhdP* locus
yhdP3	tcaTTGCGCTTTTTCTTTACGCGGTTGGCGC	

Supplemental Movie 1. Fluorescence time-lapse microscopy video tracking the signal of sfGFP-YhdP at 2-minute intervals over 80 minutes. The movie displays each frame sequentially at the speed of 4 frames per second.

Supplemental Movie 2. Fluorescence time-lapse microscopy video tracking the signal of sfGFP-TamB at 2-minute intervals over 110 minutes. The movie displays each frame sequentially at the speed of 4 frames per second.

Supplemental Movie 3. Fluorescence time-lapse microscopy video tracking the signal of sfGFP-YdbH at 2-minute intervals over 110 minutes. The movie displays each frame sequentially at the speed of 4 frames per second.

Supplemental Movie 4. Fluorescence time-lapse microscopy video tracking the signal of sfGFP-DedD at 2-minute intervals over 110 minutes. The movie displays each frame sequentially at the speed of 4 frames per second.

Supplemental references

1. Ruiz, N., Davis, R.M. and Kumar, S., 2021. YhdP, TamB, and YdbH are redundant but essential for growth and lipid homeostasis of the Gram-negative outer membrane. *mBio*, *12*(6), pp.e02714-21.

2. Wu, T., McCandlish, A.C., Gronenberg, L.S., Chng, S.S., Silhavy, T.J. and Kahne, D., 2006. Identification of a protein complex that assembles lipopolysaccharide in the outer membrane of Escherichia coli. *Proceedings of the National Academy of Sciences*, 103(31), pp.11754-11759

3. Guzman, L.M., Belin, D., Carson, M.J. and Beckwith, J.O.N., 1995. Tight regulation, modulation, and high-level expression by vectors containing the arabinose pBAD promoter. *Journal of bacteriology*, *177*(14), pp.4121-4130.
